# Amoeboma in a Saudi resident: a case report

**DOI:** 10.1099/jmmcr.0.005032

**Published:** 2016-06-10

**Authors:** Sanaa Al Rehily, Reham Kaki, Fahad Al Ghamdi, Dalia El-Hossary

**Affiliations:** ^1^​ Department of Medicine, King Abdulaziz University, Jeddah, Saudi Arabia; ^2^​ Department of Pathology, King Abdulaziz University, Jeddah, Saudi Arabia; ^3^​ Clinical and Molecular Microbiology Laboratory, King Abdulaziz University Hospital, Jeddah, Saudi Arabia; ^4^​ Department of Medical Microbiology and Immunology, Faculty of Medicine, Zagazig University, Egypt

**Keywords:** *Entamoeba histolytica*, amebic liver abscess, ameboma, hepatic abscess, colonic infection, metronidazole

## Abstract

**Introduction:**

Amoebiasis is the third most frequent cause of mortality after malaria and schistosomiasis. In developed countries, amebiasis is also seen in migrants who have travelled to endemic areas. The factors responsible for its progression from intestinal amebiasis to an amebic liver abscess are not fully understood.

**Case presentation:**

A 54-year-old man presented with abdominal pain, fever and diarrhoea. Laparotomy confirmed an inflammatory mass involving the right colon, and he underwent a right hemicolectomy. He later developed abdominal distenstion due to an amoebic liver abscess and died from secondary nosocomial bacterial infection and surgical complications.

**Conclusion:**

Amoeboma is an uncommon manifestation of amoebiasis, and can mimic both carcinoma and inflammatory bowel disease; so, distinguishing between these two conditions is the key to providing appropriate therapy. Hepatic amoebiasis is the most common extraintestinal disease of invasive amoebiasis. This clinical report presents a case of an uncommon parasitic disease in Saudi Arabia and discusses the difficulties encountered while attempting to establish the correct diagnosis. Hence, a high index of suspicion is crucial for diagnosing *Entamoeba histolytica* to avoid unnecessary surgery and further complications.

## Introduction

Amoebiasis, caused by *Entamoeba histolytica,* is rare in Saudi Arabia, but it is sometimes observed in migrants from South Asia. Hence, it should always be included as one of the differential diagnoses of acute abdomen and a colonic mass (Zaglool *et al*., 2011). Among all the parasitic diseases, amoebiasis is the third most frequent infectious disease related cause of mortality after malaria and schistosomiasis (Neghina *et al*., 2008). In developed countries, amoebiasis is also seen in migrants who have travelled to endemic areas (Freedman *et al*., 2006). An amoebic liver abscess is the most common extraintestinal manifestation of amoebiasis. Approximately 90 % of patients with a liver abscess are male (Acuna-Soto *et al*., 2000). Amoebae establish hepatic infection by ascending into the portal venous system (Liu & Crawford, 2004). The factors playing a role in the progression from intestinal amoebiasis to an amoebic liver abscess are not fully understood, but those that affect cell-mediated immunity may increase the risk of *E. histolytica* infection, which result in an invasive form of the disease with liver involvement (Park *et al*., 2007). Amoeboma formation occurs in only about 1.5 % of patients (Misra *et al*., 2006). Colonic amoeboma is accompanied by an amoebic liver abscess that can be misdiagnosed as metastatic carcinoma of the colon (Stockinger, 2004).

The purpose of this report is to describe and highlight the importance of serological techniques for the early detection of complications due to *E. histolytica* infection.

## Case report

A 54-year-old man presented to our hospital with a 10-day history of diffuse, dull, aching, non-radiating abdominal pain that was associated with a documented high-grade fever and bloody diarrhoea (3–4 episodes/day). He had travelled to Pakistan and, during the preceding three months, he had noticed an increase in the frequency and looseness of his stool. The patient had no history of diarrhoea before this. He was previously diagnosed with hypertension but had no history of diabetes mellitus, tuberculosis, smoking or alcohol consumption. The patient’s HIV and hepatitis B surface antigen status were negative. No significant medical or surgical history was present.

On physical examination, the patient looked unwell and had shortness of breath (respiratory rate: 35) with desaturation [peripheral capillary oxygen saturation (SpO_2_) 88 %]. The abdomen was mildly tender with no palpable abdominal mass. Hepatomegaly was present (the liver measured 17 cm), but no splenomegaly. On digital rectal examination, the rectum was empty, and the examining finger was tinged with blood and mucus. Blood tests showed marked neutrophilic leukocytosis (white cell count: 24.6 × 10^9−L^) and mildly deranged liver function tests. In the emergency ward, he developed renal impairment and was immediately started on dialysis. Because of SpO_2_ desaturation, the patient received bilevel positive airway pressure (BiPAP), and was moved to the intensive care unit (ICU).

## Investigations

The general surgeon was consulted for a differential diagnosis, including perforated peptic ulcer disease and a neoplasm. Contrast-enhanced computed tomography (CT) of the abdomen was planned. Computerized tomography (CT) scan of the abdomen showed a diffuse irregular circumferential thickening of the caecum, and the right ascending colon reached the hepatic flexure, measuring around 3.7 cm. This was associated with a significant narrowing of the lumen. Focal air pockets were seen at the wall of the right colon that possibly represented a sealed perforation. The rest of the large bowel loops had totally collapsed, and the small bowel loops were dilated with multiple levels of air fluid, suggesting sub-acute intestinal obstruction. There was generalized fat stranding, which was more prominent in the right iliac fossa and the area of the greater omentum with multiple heterogeneous soft tissues. These may have been an enlarged lymph node instead of omental deposits. The liver was enlarged and measured 18 cm with two hypodense non-enhancing lesions, the largest of which measured 4.6 × 4.4 × 5 cm.

On the next day of hospitalization, the patient showed deterioration in health and eventually developed sepsis.His level of consciousness using the Glasgow Coma Scale (GCS) was 10/15. So he required immediate clearing of his airways. He was empirically administered broad-spectrum antibiotics like parenteral vancomycin 7.5 mg kg^−1 ^every 48 h (renally adjusted dose) and parenteral meropenem 500 mg twice a day (renally adjusted dose) while waiting for the results of blood cultures that were performed using an automated blood culture system (BacT/Alert; Organon Teknika, USA). Five millilitres of blood or peritoneal fluid were inoculated into aerobic and anaerobic bottles that were loaded with BacT/Alert blood culture system and incubated for a maximum of five days. Smears were prepared from the positive culture bottles and were evaluated using Gram staining. The positive specimen was subsequently sub-cultured onto plates with blood agar, MacConkey agar, chocolate agar, and Sabouraud agar (SDA; Saudi Prepared Media Laboratories, Riyadh, Kingdom of Saudi Arabia), which were incubated for 18–24 h. The stained smears revealed the presence of gram-negative bacilli. Further identification and antimicrobial susceptibility testing were performed using a VITEK® 2 system (bioMerieux, France) according to the manufacturer’s instructions. In case of *Acinetobacter baumannii*, the minimum inhibitory concentration (MIC) of amikacin and meropenem were determined using E-test strips (bioMerieux, France).

Culture bottles positive for yeast cells were cultured onto SDA, and the yeasts were identified using VITEK MS on the same day of sufficient growth on SDA; the identification (ID) and antifungal susceptibility were then confirmed by using the VITEK ® 2.

## Diagnosis

After reviewing the clinical data and radiological imaging, a provisional diagnosis of an obstructing right-sided colonic carcinoma with liver metastases was made.

## Treatment

An emergency laprotomy showed that the right colon was perforated by multiple abdominal collections, including two liver abscesses. A right hemicolectomy was performed, including removal of a part of the ileum, with an ileostomy. The two liver abscesses were drained and biopsied.

On the fourth day of hospitalization (i.e., the post-operative period), the patient’s health deteriorated again, and he showed unstable vital signs with abdominal distension. The patient wasre-evaluated and all work-up was repeated. Based on the radiographic new bilateral chest infiltrates and positive tracheal aspirate for multidrug-resistant strains in culture growths, he was diagnosed to have had ventilator-associated pneumonia. The results of *A. baumannii, Pseudomonas aeruginosa*, and *Stenotrophomonas maltophilia* susceptibility testing are shown in Table 1. Based on these findings, parenteral colistin 2 million every 36 h (renally adjusted dose) and co-trimoxazole 960 mg every 24 h were started. Paracentesis was performed, and the culture was positive for *Candida tropicalis* and *S. maltophilia* (Table 1). Caspofungin was started initially, then switched to fluconazole once sensitivity was confirmed. An urgent abdominal CT confirmed leakage at the anastomosis site with an increased number of hepatic lesions that mostly represented newly developed abscesses. Further re-exploration was undertaken. The previous anastomosis was removed, and new loop ileostomy was performed.

**Table 1.** Bacterial culture results from different sites, along with susceptibility testing

Culture	Site	Species identified	Antimicrobial	Sensitivity
**Fluid culture**	Peritoneal	*Candida tropicalis*	Amphotericin	S^†^
			Caspofungin	S
			Flucytosine	S
			Fluconazole	S
**Fluid culture**	Peritoneal	*Stenotrophomonas maltophilia*	Levofluxacin	S
			Sulfamethoxazole-trimethoprim	S
**Sputum culture**	Tracheal aspirate	*Acinetobacter baumannii*	Meropenem	R^*^
			Piperacillin/tazobactam	R
			Ciprofloxacin	R
			Colistin	S
			Cefepime	R
			Imipenem	R
			Tigacycline	R
**Sputum culture**	Tracheal aspirate	*S. maltophilia*	Sulfamethoxazole-	S
			trimethoprim Levofluxacin	S
**Sputum culture **	Tracheal aspirate	*Pseudomonas aeruginosa*	Meropenem	S
			Piperacillin/tazobactam	S
			Ciprofloxacin	S
			Cefepime	S
			Gentamycin	S
			Imipenem	R
			Ceftazidime	S

†S, sensitive, R*, resistance

**Fig. 1.**
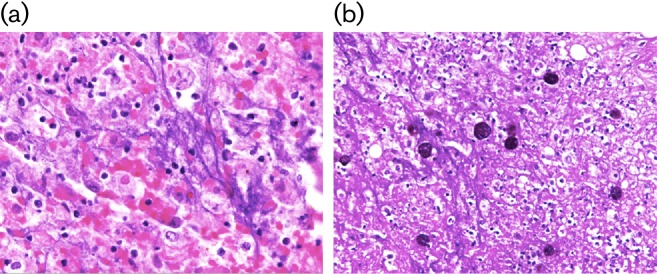
Liver abscess with *Entamoeba histolytica* trophozoites. (a) Round to oval parasites surrounded by halos on a debris-filled background (hematoxylin and eosin stain; 200× power). (b) The parasites changed from pink to magenta (PAS stain; 100× power).

The histopathology report showed evidence of a liver abscess containing round to oval parasites surrounded by halos on a debris-filled background. The parasites exhibited large, round eosinophilic nuclei and abundant vacuolated cytoplasm that changed from pink to magenta with periodic acid-Schiff (PAS) special stain, a technique to enhance detection of E. histolytica trophozoites; (Fig. 1) (Ning *et al.*, 2012). The morphology of these parasites was consistent with *E. histolytica* trophozoites.

A subsequent serological test to detect antibodies specific to *E. histolytica* (Cellognost* Amoebiasis, Siemens Healthcare Diagnostics Products GmbH, Marburg, Germany) was performed. This test is an indirect hemagglutination test used for quantitative determination of antibodies specific to *E. histolytica,* and has been used for testing of human serum obtained from patients with invasive amoebiasis. In this case, the test revealed an elevated titre of *E. histolytica* immunoglobulin G (IgG) antibody (amoeba Ab titre = 1 : 1024; Fig. 2). After eight days from initial presentation, the patient was started on oral metronidazole 750 mg every 8 h. There was no evidence of neoplastic disease or atypical cells on the histopathological report; amoebic trophozoites were stained using periodic acid– schiff (PAS), confirming the diagnosis of *Entamoeba*.

**Fig. 2.**
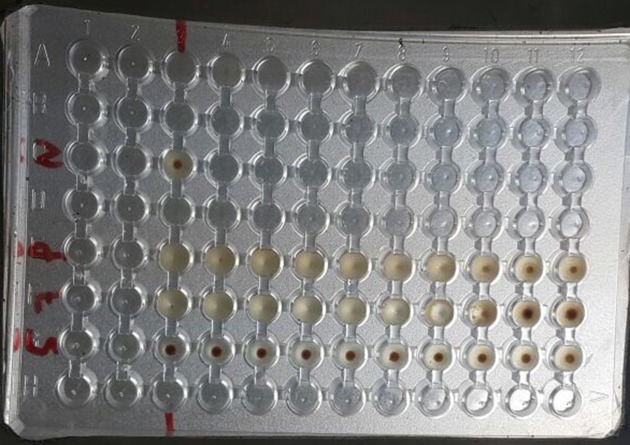
Hemagglutination assay, indirect hemagglutination microtitration plate showing "N", negative control and "P", positive control. Sample number 5 is negative and sample number 7 is the sample of our patient with a positive titer of 1/1024.

## Outcome and follow-up

There was an uneventful recovery for 11 days. However, on day 12 after admission, his health deteriorated again, and he developed bacteraemia due to central line infection with multidrug-resistant *A. baumannii* (Table 1). The patient did not recover from the septic shock and continued to deteriorate with hemodynamic instability, despite receiving high doses of inotropes. Due to his critical condition, he was not a candidate for surgery. Unfortunately, the patient died on day 16 of admission, due to nosocomial intra–abdominal infection and surgical complications.

### Discussion

Approximately 80 % of patients with an amoebic liver abscess show relatively rapid development of symptoms (typically within 2–4 weeks), including fever, cough, and a constant, dull aching abdominal pain in the right upper quadrant or epigastrium (Hoffner *et al*., 1999; Katzenstein *et al*., 1982; Haque *et al*., 2003; Knobloch & Manweiler, 1983). An amoebic liver abscess is rare, and is seen almost exclusively in immigrants or travellers to endemic areas. An estimated <1 % of patients with amoebic colitis develop an amoebic liver abscess (Skappak *et al*., 2014). The abscess is usually single, and in 80 % of cases, it is found in the right lobe of the liver (Adams & Macleod, 1977; Hoque *et al*., 2014). Uncommonly, patients with amoebic hepatic abscesses may also have localized colonic infection resulting in a mass of granulation tissue forming an amoeboma that may mimic colonic cancer (vanSonnenberg *et al*., 1985). Serological tests represent the cornerstone of the diagnosis of an amoebic liver abscess and are an important adjunct in the diagnosis of intestinal amoebiasis. These tests are positive in 95 % of patients with amoeboma but in only 60 % of those with amoebic colitis (Haque *et al.*, 2003). Many serological assays have been widely used for the detection of antibodies, all of which have sensitivity >93 % and specificity >90 % and are superior to stool ova and parasite examination (Parija & Karki, 1999; del Carmen Sanchez-Guillen *et al*., 2000; Haque *et al*., 2003). However, these tests have some limitations as they are unreliable in confirming the diagnosis in endemic countries (Fotedar *et al*., 2007). However, there is one particular enzyme-linked immunosorbent assay (ELISA) made with antigens and preserved without using enzymatic inhibitors that has serodiagnostic value in the diagnosis of acute amoebic liver abscess, even in populations that have antibodies against amoebas living in endemic zones of amoebiasis (Flores *et al*., 2016).

In this case, radiological imaging of the liver lesions was not typical of amoebic abscesses, which created the diagnostic dilemma. The main differential diagnoses considered were pyogenic liver abscess and metastatic colonic cancer. Surgical resection was performed because of the possibility of colonic malignancy. During laparotomy, an inflammatory mass involving the right colon was confirmed, and a right hemicolectomy was performed. Nitroimidazoles, particularly tinidazole, are the mainstay therapy for invasive amoebiasis (Abd Alla *et al*., 2007); metronidazole is the most commonly available drug for treatment. The cure rate with this therapy is >90 % (Lasserre* et al*., 1983). Metronidazole is well absorbed in the gastrointestinal tract. Hence, intravenous therapy offers no significant advantage when the patient is administered orally and has no major defect in small bowel absorption. Superimposed bacterial infection of an amoebic liver abscess has occasionally been observed (both before and as a complication of the drainage). Therefore, broad-spectrum antibiotics, surgical drainage, or both should be added to the treatment regimen if the patient does not have a prompt response to nitroimidazole.

Surgery is rarely required and is indicated only in cases of diagnostic uncertainty or if toxic megacolon occurs. Therapeutic aspiration of an amoebic liver abscess is occasionally required as an adjunct to antiparasitic therapy. Drainage of the abscess should be considered in patients that show no clinical response to drug therapy within 5–7 days of its administration or in those with a high risk of abscess rupture, which is defined by a cavity with a diameter of >5 cm or the presence of lesions in the left lobe (vanSonnenberg *et al*., 1985).

In this case, surgery could have been avoided if a preoperative diagnosis of amoebiasis was made. However, the clinical presentation and radiological imaging were not typical. A few similar case reports have been reported where colonic amoebiasis mimics an obstructing right-sided colonic carcinoma with liver metastases. Amoebiasis is rare in the Saudi Arabian population, but is sometimes seen in migrants from South Asia. When patients present with a recent history of dysentery and travel to areas where amoebiasis is endemic, this should prompt further investigations including serological tests to exclude amoebiasis. An increased awareness and a high index of suspicion is crucial for correct diagnosis and management to avoid unnecessary surgery and prevent further complications.
